# Atomic Force Microscopy Protocol for Measurement of Membrane Plasticity and Extracellular Interactions in Single Neurons in Epilepsy

**DOI:** 10.3389/fnagi.2016.00088

**Published:** 2016-05-04

**Authors:** Xin Wu, Mariappan Muthuchamy, Doodipala Samba Reddy

**Affiliations:** ^1^Department of Neuroscience and Experimental Therapeutics, Texas A&M Health Science Center College of MedicineBryan, TX, USA; ^2^Department of Medical Physiology, Texas A&M Health Science Center College of MedicineBryan, TX, USA

**Keywords:** atomic force microscopy, ECM protein, integrins, epilepsy, neuronal plasticity

## Abstract

Physiological interactions between extracellular matrix (ECM) proteins and membrane integrin receptors play a crucial role in neuroplasticity in the hippocampus, a key region involved in epilepsy. The atomic force microscopy (AFM) is a cutting-edge technique to study structural and functional measurements at nanometer resolution between the AFM probe and cell surface under liquid. AFM has been incrementally employed in living cells including the nervous system. AFM is a unique technique that directly measures functional information at a nanoscale resolution. In addition to its ability to acquire detailed 3D imaging, the AFM probe permits quantitative measurements on the structure and function of the intracellular components such as cytoskeleton, adhesion force and binding probability between membrane receptors and ligands coated in the AFM probe, as well as the cell stiffness. Here we describe an optimized AFM protocol and its application for analysis of membrane plasticity and mechanical dynamics of individual hippocampus neurons in mice with chronic epilepsy. The unbinding force and binding probability between ECM, fibronectin-coated AFM probe and membrane integrin were strikingly lower in dentate gyrus granule cells in epilepsy. Cell elasticity, which represents changes in cytoskeletal reorganization, was significantly increased in epilepsy. The fibronectin-integrin binding probability was prevented by anti-α5β1 integrin. Thus, AFM is a unique nanotechnique that allows progressive functional changes in neuronal membrane plasticity and mechanotransduction in epilepsy and related brain disorders.

## Introduction

Epilepsy affects ~3 million people in the U.S. Currently there is no cure to epilepsy, nor a way of preventing epileptogenesis, the process by which a normal brain develops epilepsy due to a variety of risk factors including injury or genetic predispositions. Extracellular matrix (ECM) proteins in the brain are produced and secreted by neuronal cells. During neural development, ECM molecules play a critical role in neuroplastic events in the hippocampus, a key region involved in epilepsy. Neuroplasticity is described as the adaptive changes to extrinsic or intrinsic encounters, such as epileptogenic injury or brain injury by the nervous system (Browne and Holmes, 2001). Integrins are a large family of transmembrane heterodimeric receptors (α,β subunits) that provide a connection between ECM and the intracellular focal adhesion complex (FAC) molecules including non-receptor tyrosine kinases (NRTKs) focal adhesion kinase (FAK) and Src, as well as cytoskeleton proteins, including talin and actin (Wu et al., 1998, 2001; Pinkstaff et al., 1999; Wu and Reddy, 2012).

Neuronal membrane elasticity, plasticity, and related dynamics can greatly affect the neuronal responses to network discharges and epileptic seizures that can be studied by sophisticated atomic force microscopy (AFM). The AFM, invented in 1986 (Binnig et al., 1986), has emerged to be a powerful instrument for studying ligand-receptor and cell-cell interactions, as well as the mechanical properties of living cells in the neuronal and other biological research. The AFM passively senses the localized forces between AFM scanning probe and molecules on the cell surface under unique three-dimensional (3D) movements through an extremely fine sharp cantilever tip (size-nanometer). AFM can provide information regarding adhesion/binding force between molecules up to piconewton (pN) and high resolution 3D surface structural imaging up to nanometer. In addition, AFM can directly measure the association between cell mechanical properties (e.g., elasticity) and intracellular cytoskeleton proteins and organelles. Future direction in these mechanotransduction studies points to the combination of AFM technology with patch-clamp technique, confocal microscopy, and total internal reflectance fluorescence for probing cellular structure, function and signaling (Kassies et al., 2005; Trache and Meininger, 2005; Sun et al., 2009; Wu et al., 2012a). In this study, we describe an optimized AFM protocol and its application for measurement of membrane plasticity and mechanical dynamics of individual hippocampus neurons in mice with chronic epilepsy.

## Materials and equipment

### Neuronal cell isolation materials

All equipment and materials for hippocampus slice preparation, single cell isolation and AFM probe coating preparation are commercially available (e.g., VWR and Fisher Scientific). In order to easily duplicate this experiment, equipment and materials have been listed with their company's name, comments, as well as catalog number or model number in the Table 1. All chemicals, except if specifically stated, were obtained from Sigma-Aldrich. ECM protein and antibody were purchased from BD Biosciences.

**Table 1:** **Materials and reagents for neuronal cell isolation**.

**Name**	**Company**	**Catalog/Model**	**Qt**.	**Comments**
Dissecting microscope	Nikon	SMZ 647	1	Micro-dissecting subfields of CA1, CA3 and dentate gyrus (DG)
Vibratome with 900 Refrigeration System	Leica Microsystems, Inc, Bannockburn, IL	1500	1	Hippocampus tissue slices cutting
Water bath	Thermo-Fisher Scientific, Waltham, MA	2876	1	Keep the temperature stable for slices and single cell isolation
Mixed gas tank (95% O2 + 5% CO2)	Local company	Medical grade	1	Oxygenizing physiological solution
Glass Bottom Dish	In Vitro Scientific or WillCo-Dish	D60-30-1-N		60 mm dish with 30 mm bottom well, No. 1 Glass (0.13-0.15 mm). used on AFM stage
Culture dish (60 mm)	VWR	25,382-381	1	Temporary storage of brain tissue during dissecting
Brain slice keeper	Scientific Systems Design, Inc., Ontario L5T 2J5 Canada	BSK4	1	Submerged slice pre-incubator, four rings
Scalpel Handle No. 3	Harvard Apparatus	72-8350	1	Skin cut
Scalpel Blades No. 10, Sterile	Harvard Apparatus	72-8360	n/a	Skin cut
Micro Friedman Rongeur; Curved, 1.3mm Jaw, Width; 5.5″ Length	Roboz Surgical Store	RS8303	2	Isolating tissue
Bone Pliers	Fine Science Tools	16,025-14	1	Skull cut
Nickel/Stainless Steel Spatula/Spoon	VWR	47,0149-440	1	Transferring the brain tissue
Spatula with Tapered Blade	Corning	3003	n/a	Transferring the brain tissue
Kuehne Forceps 4″ Straight Flat Jaw	Roboz Surgical Store	RS-8261	2	Isolating tissue
Feather, Double Edge Carbon Steel Blades	Ted Pella, Inc	121-9	n/a	Brain cut
Electric Shaver	Commercial available	N/A	1	Animal hair cut
Pasteur glass fire pipette	Fisher Scientific	13-678-4	1	Single cell isolation
Contrad 70	Fisher Scientific	04-355	1	Cleans glass, plastic, ceramic and ferrous metals; For soaking, scrubbing, or ultrasonic cleaning
Kynurenic acid	Tocris Bioscience, Minneapolis, MN	0223	1	An excitatory amino acid receptor antagonist at AMPA, NMDA, and Kainate glutamate receptors
Protease XXIII	Sigma-Aldrich, St. Louis, MO	P4032	1	For cell isolation, if use
Polyethylene glycol	Sigma-Aldrich, St. Louis, MO	P-5413	1	AFM probe coating
Fibronectin	BD Biosciences	356,008	1	Extracellular matrix protein
Hamster Anti-mouse CD49e (HMα5-1), MAB	BD Biosciences	553,350	1	Integrin antibody

### Atomic force microscope (AFM) configuration (Figure 1)

**Figure 1:**
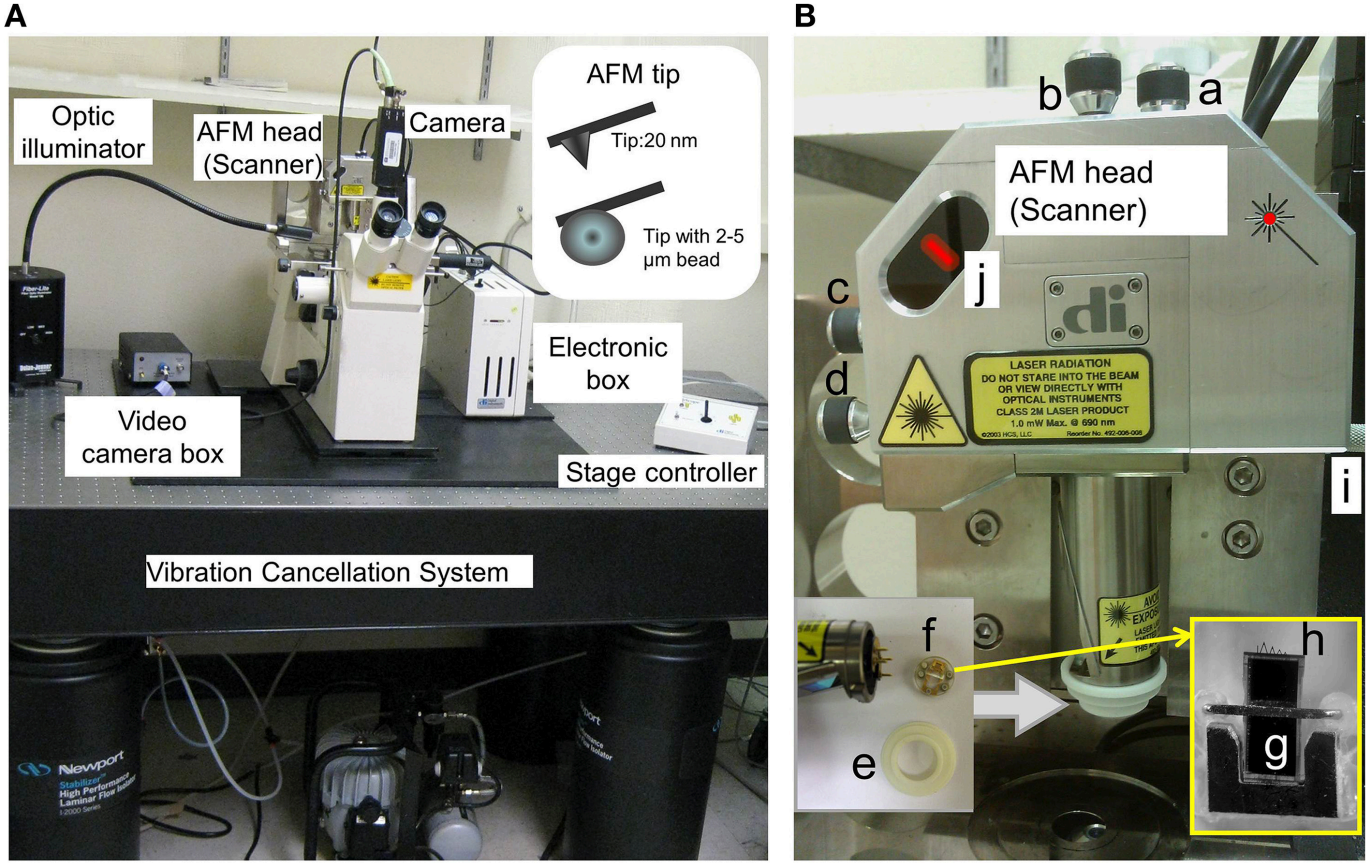
**AFM hardware configuration**. **(A)** AFM head (or Scanner) in the stand is shown. The standard open-loop AFM head contains piezoelectric scanner tube, laser beam, and quadrature photodiode detector. It scans areas about 90 μm in *x-y-* and 6 μm in *z*-axis. A sharp AFM probe tip (nanometers in diameter) and AFM probe with bead (micrometers in diameter) are shown in the insert. **(B)** “a and b,” screws to fine-tune laser position on the back of AFM probe cantilever; “c and d,” screws to fine-tune laser position in the photodiode detector; “e,” O-ring to secure the fluid holder from the liquid; “f,” AFM probe holder. AFM probe includes supporting chip, cantilever and the tip. “g,” supporting chip in the AFM probe in the cartridge of the AFM holder (f); “h,” “V”-shaped cantilevers in the AFM probe, which contain the pyramidal tips in the end, and one pyramidal tip is shown in **(A)** insert and Figure 2; “I,” Screws in the AFM stand for AFM Scanner (loose the screw to secure the AFM head and tighten the screw to release the AFM head); and “j,” laser position and intensity indicators (red color bar) in the AFM Scanner.

1. Windows computer for recording (minimum Intel Pentium 4, 1 GB memory and 500 GB hard drive)
2. Axiovert 100 TV inverted microscope (Carl Zeiss, Jena, Germany) with motorized stage controlled by BioScope stage controller (Digital Instruments, Santa Barbara, CA. (Bruker Corporation Now)). Carl Zeiss 32x objective (air, N.A. = 0.4).
3. Video camera (Pulnix, model TM 34KC, Yokohama, Japan)
4. Newport Stabilizer Vibration Control System (I-2000 series, Newport, Irvine, CA)
5. AFM hardware and software (Example: Bioscope Model IIIA, Bruker Corporation)
6. AFM cantilevers: (a) silicon nitride with pyramidal shaped tip has mean spring constant at 14.4 ± 0.6 pN/nm and diameter at less than 40 nm (Part #:0010, Bruker Corporation. (model #: MLCT-AUHW, Santa Barbara, CA)); or (b) borosilicate beads labeled with biotin has mean spring constant at 0.01 N/m and diameters at 2 to 5 μm (Cat#:PT.Broo.Bio.SN, Novascan Technologies, Inc, Ames, IA. Figure 1A, insert).

### Solutions

1. Artificial cerebrospinal fluid (ACSF) buffer was composed of (in mM): 126 NaCl, 2 MgCl_2_, 3 KCl, 1.25 NaH_2_PO_4_, 2 CaCl_2_, 26 NaHCO_3_, 11 glucose and 0.3 kynurenic acid (pH = 7.35-7.40 equilibrated by a gas mixture 95% O_2_ + 5% CO_2._Osmolarity = 310 ± 5 mOsm/kg).
2. The physiological saline solution (PSS) for AFM recording was composed of (in mM): 140 NaCl, 2 MgCl_2_, 3 KCl, 2 CaCl_2_, 10 HEPES, and 16 glucose (pH = 7.4 with NaOH. Osmolarity = 320 ± 5 mOsm/kg).

## Methods

### Brain slice preparation

1. Transverse hippocampal slices (thickness = 400 μm) were prepared from adult C57BL/6J strain male mice (2–4 months old) and were utilized for dissociation of neuronal cells such as dentate gyrus granule cells (DGGCs) (Reddy and Jian, 2010; Wu et al., 2013; Carver et al., 2014). All procedures were performed in strict compliance with the guidelines of National Institutes of Health Guide for the Care and Use of Laboratory Animals under a protocol approved by the university's Institutional Animal Care and Use Committee.
2. Mice were anesthetized with isoflurane (Notes 1 in Box 2). Then, the brain was rapidly removed and kept with 4°C in ACSF bubbled with carbogen gas (95% O_2_ + 5% CO_2_) (Notes 2). Animals were euthanized after decapitated.
3. Several 400 μm slices were cut with a Vibratome. Brain slices were equilibrated at 23 ± 1°C in ACSF on a mesh surface in a small beaker or Brain Slice Keeper in a water bath continuously with carbogen gas (Notes 3).

### Dissociation of subfields of hippocampus

1. The hippocampi including the DG region (similar protocol for CA1 or CA3 region) were microdissected carefully under the dissecting microscope (Wu et al., 2013).
2. The isolated subfield slices were then incubated in Brain Slice Keeper in ACSF for 1 h at 24°C. For non-enzyme procedure, skip next step 3.2.3.
3. The slices were transferred into ACSF with protease XXIII (3 mg in 1.0 ml), and incubated for 24 ± 1 min at 23 ± 1°C. Then, the DG slices were washed by 1 ml ACSF for 3 times.
4. Triturated the slices to separate the cells. (Notes 4). The suspended cells were carefully pipetted into the glass bottom dish for AFM (Wu et al., 2013).

### The principle of AFM

**Figure 2:**
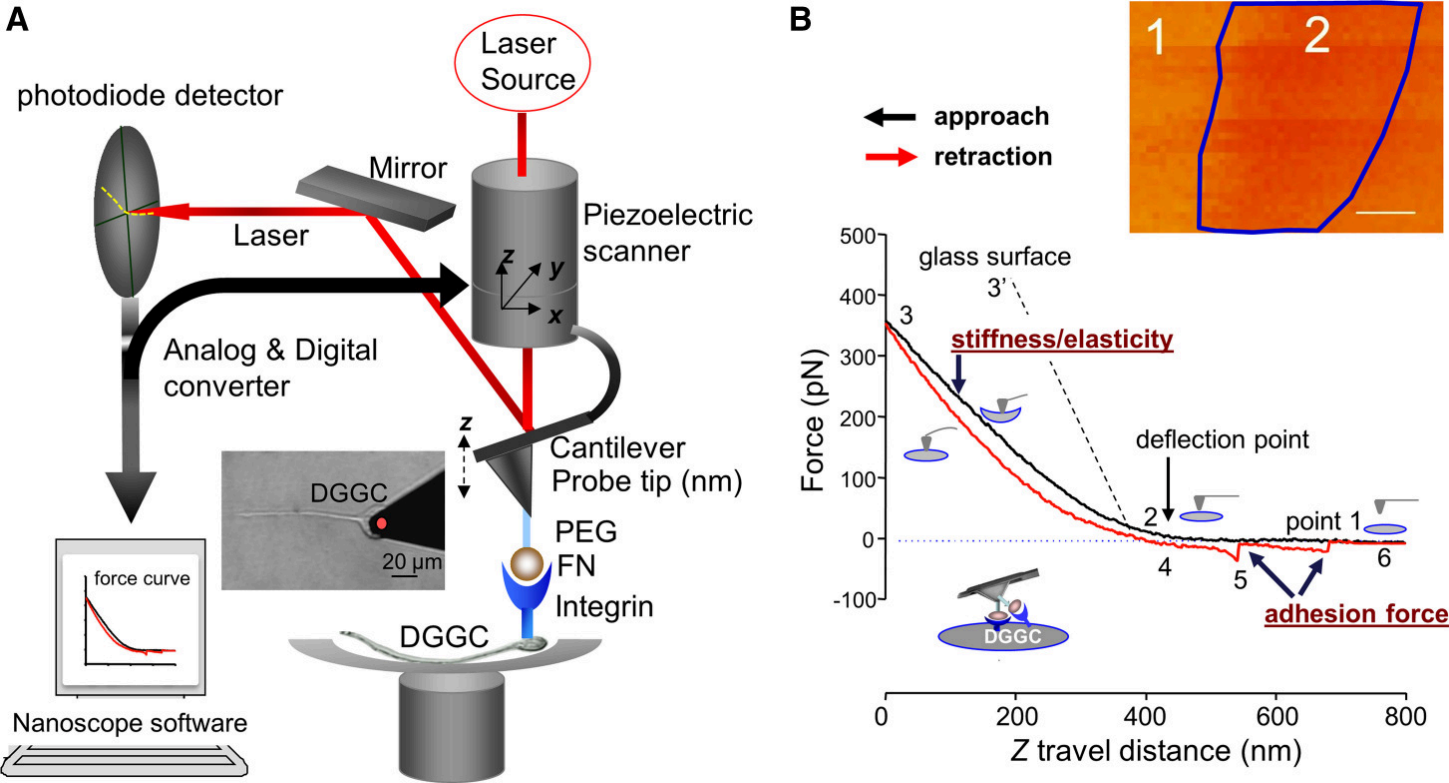
**Diagram representation of the AFM and constant force curve**. **(A)** AFM holder is rigidly connected to a 3-dimensions (3D or x-y-z-) piezoelectric component. The deflection of the cantilever will be detected by a laser beam and displayed position changes in the segmented photodiode while AFM tip moving at cell surface with *xyz*-axes. The NanoScope software is a feedback piezo-control system. It will control and record the cantilever deflection and the interaction forces. A PEG-ECM protein fibronectin (FN)-coated AFM probe and integrin receptor is also indicated. A force curve or 3-D image will be collected by the system. The AFM probe attaching a DGGC is showed in the inserted image. **(B)** Original force curves data recorded from FN-coated AFM probe on DGGC. FN-coated AFM probe is controlled to repeatedly (z-axis movement: 800 nm and scan rate = 0.5 Hz) approach/attaching (black trace) and retract/withdrawal (red trace) from single DGGC at a given “*x” and “y”*-axes. The stages of attaching and withdrawal are showed in the points 1–6. The insert image shows force volume imaging for mapping elastic features over axon hillock of living DGGC. While simple forces curve **(B)** records the force felt by the tip as it approaches and retracts from a point on the cell surface, the study of cellular mechanics often requires characterization of the distribution or variance of these forces over 3-D structures. A force volume contains an array of force curves over the selected cell area. Each force curve (z-axis movement) is measured at a unique x–y position in the area, and force curves from an array of x–y points are combined into a 3D array, or “volume” of force data. Dark region (label “2” within blue frame) represents less stiffness than light regions (label “1”). The quantization of normalized mean intensity will be used to analyze the difference of elasticity in different regions or different cells. Bar = 100 nm in **(B)**. PEG, Polyethylene glycol; ECM, extracellular matrix protein.

Conventional optical microscopy can examine live sample morphology and remodeling following time, as well as proteins specificity and density if combined with fluorescence. AFM is based on a laser tracking the deflection of a sharp nanometer cantilever tip, while simultaneously sensing the local force, energy, loading, and stiffness between the molecule on the tip and sample surface in real-time (Figure 2A). The advantages of AFM include: (1) the data are recorded by sensing the sample surface and underneath without using the light, even though most AFM systems are integrated with optical imaging; (2) the nano-sensor on the tip is able to probe single molecular events in living cells. It is the only tool that enables us to visualize the sub-molecular resolution of the major and minor grooves of the DNA double helix under physiological conditions. This is essential for considering the structure-function relationship of biomolecular systems *in vivo* and for *in situ* analysis of DNA-based nano-devices (Ido et al., 2013; Pyne et al., 2014); (3) the probe serves as nano-manipulation tool for pressing, pulling and rolling on cell surface; and (4) that AFM is the only microscopic method available to provide both functional and structural information at a high resolution. However, AFM has following limitations: (1) the resolution will depend on the tip radius and cantilever spring constant. The physical AFM probe used in imaging is not always associated with the cell geometrical features. Consequently, the AFM image will not reflect true cell topography. In addition, a blunt or contaminated tip may cause imaging deformations, especially in freshly isolated cells. These types of artifacts can be easily avoided by using sharp clean AFM tips with more sensitive spring constant cantilevers, as well as selecting high resolution and slower scanning on the cell surface; (2) the measurement is only at the apical cell surface; (3) evaluations of stiffness and imaging are occasionally difficult because of the irregular topography and complex mechanical properties in cell membrane; and lastly, (4) it requires open configuration for the research on living cell and very good vibration or acoustic insulation to avoid the noises.

AFM tips/probes come in different shapes and sizes, such as sharp pyramidal nanometer tip made from silicon or silicon nitride or glass/polystyrene micrometer beaded tips (Figure 1A, insert). The AFM probe sensitivity depends on the property of the flexible cantilever. The tip diameter governs the spatial resolution, the smaller in the apex size of AFM probe and the higher the resolution in imaging and force measurement. The closed loop feedback piezo-control system in the AFM program permits for the monitoring of binding forces between the tip and cell surface, and through digital/analog converter, and also controls the piezoelectric scanner that monitors movement of AFM probe on the surface of the cell. The movement of the laser beam that detects deflections of cantilever will be noticed by segmented photodiode detector (Figure 2A). The photodiode detector then sends back the signal to AFM program through analog/digital converter system.

### The maneuver of AFM

The AFM recording can be achieved through tapping (intermittent) and contact modes.

#### The tapping mode

During the mapping of the sample, the cantilever in AFM probe is oscillated at its resonant frequency (bouncing up and down) under an external electrical excitation, lightly “taps” on the cell surface, and contacts the surface at the bottom (*z*-axis) of each swing at each given *xy*-point. By maintaining constant oscillation amplitude, a constant tip-sample interaction is maintained and an image of the surface is obtained as described by Wu and colleagues (Wu et al., 2012b). The advantages of tapping mode include (a) allowing high nanometer resolution of samples; (b) the ability to be used for freshly isolated cells that are loosely held to the bottom of a dish; and (c) the ability to record the height of the image. The disadvantages of tapping mode are that (a) it does not offer a good image in a liquid; (b) the samples are possibly damaged and (c) slower scan speeds are needed for the tapping mode of operation, otherwise it will be too noisy in the data recorded in the liquid. Recent PeakForce quantitative nanomechanical mapping (PeakForce QNM) technique is based on tapping mode and the acquisition frequencies up to 1 kHz in liquid. PeakForce QNM simultaneously generates height, adhesion, and modulus data, whereas Tapping Mode yields only height and phase data.

#### The contact mode

In this mode, the spring constant of cantilever in the AFM probe is much less than the spring of the cell surface, so the cantilever will bend when it attaches to the cell. The force between the probe and the cell stays constant through the closed piezo-feedback loop control system and the surface image is obtained by moving the z-scanner for each *xy* point. The “height” in the image reflects the true height data of the cell. The advantages of contact mode are that it is optimal for cultured cells, faster at scanning than tapping mode, and useful for rough samples and imaging analysis as well as obtaining more fine details of the sample in the “deflection” image presented. The disadvantages are (a) these images lose the true cell height information, (b) damage or deformation can occur to soft samples by movement on the sample surface and (c) contact mode is not optimal for imaging freshly isolated cells because of loosely held to a dish bottom.

Contact mode is easier to manipulate and operate than tapping mode, and more convenient for switching between constant force and imaging mode. In this article, we describe a detailed protocol using AFM to perform integrin-extracellular matrix interactions in neuronal cells in constant force mode.

#### Constant force mode

In this mode, the AFM tip treated with ECM protein is brought into contact with the neuronal cell surface. The piezo-control system records the deflection signal of the cantilever by moving the *z* scanner over a predefined distance at each given *xy* point. In most cases, the *xy*-axis scan size is fixed, and the position of the probe is controlled in order to repeatedly contact and retract from the cell surface. The deflection signal from the cantilever tip's indentation is recorded and drawn as *z*-position vs. deflection of the cantilever tip, called as “force curve” (Figure 2B, Video 1). The indentation in the contact point is related to the shape of the tip, the cantilever spring constant and the cell mechanical properties.

While single force curves (z-axis movement, Figure 2B) record the force sensed by the tip at a point of the cell surface, the study of cellular mechanics often requires the characterization of the distribution or variance of these forces over 3-D structures. A group of force curves across a selected cell area is reconstructed into a 3D array and called *force volume*, i.e., “volume” of force data (Figure 2B, insert). Here, a force volume elasticity map was constructed from axon hillock of DGGC in control mice. The dark pixel regions (Figure 2B, label 2) represent less stiffness than the light regions (label 1). The disadvantage of force volume is that it is quite time consuming (hours needed for one 512 × 512 resolution). Current Fast-Force volume (acquisition frequencies: from < 1 Hz to 300 Hz) measurement will include data of adhesion, force modulus, stiffness, and height.

### Stepwise procedures of AFM on constant force mode

The AFM has been used to study in a wide variety of samples including biological samples (Trache et al., 2005; Wu et al., 2010a,b, 2012b; Tangney et al., 2013a). In biological samples, the AFM technique has also been successfully applied in cardiomyocytes (Wu et al., 2010a; Tangney et al., 2013a), vascular arteriolar smooth muscle cells (Sun et al., 2005), arteriolar endothelial cells (Trache et al., 2005), and neuronal cells (Parpura et al., 1993; Pasternak et al., 1995; Kirmizis and Logothetidis, 2010; Wu et al., 2012a; Spedden and Staii, 2013). In the next section, we discuss the AFM contact mode on DGGCs (Box 1).

Box 1 **Flow chart shows the general outline of contact mode.**

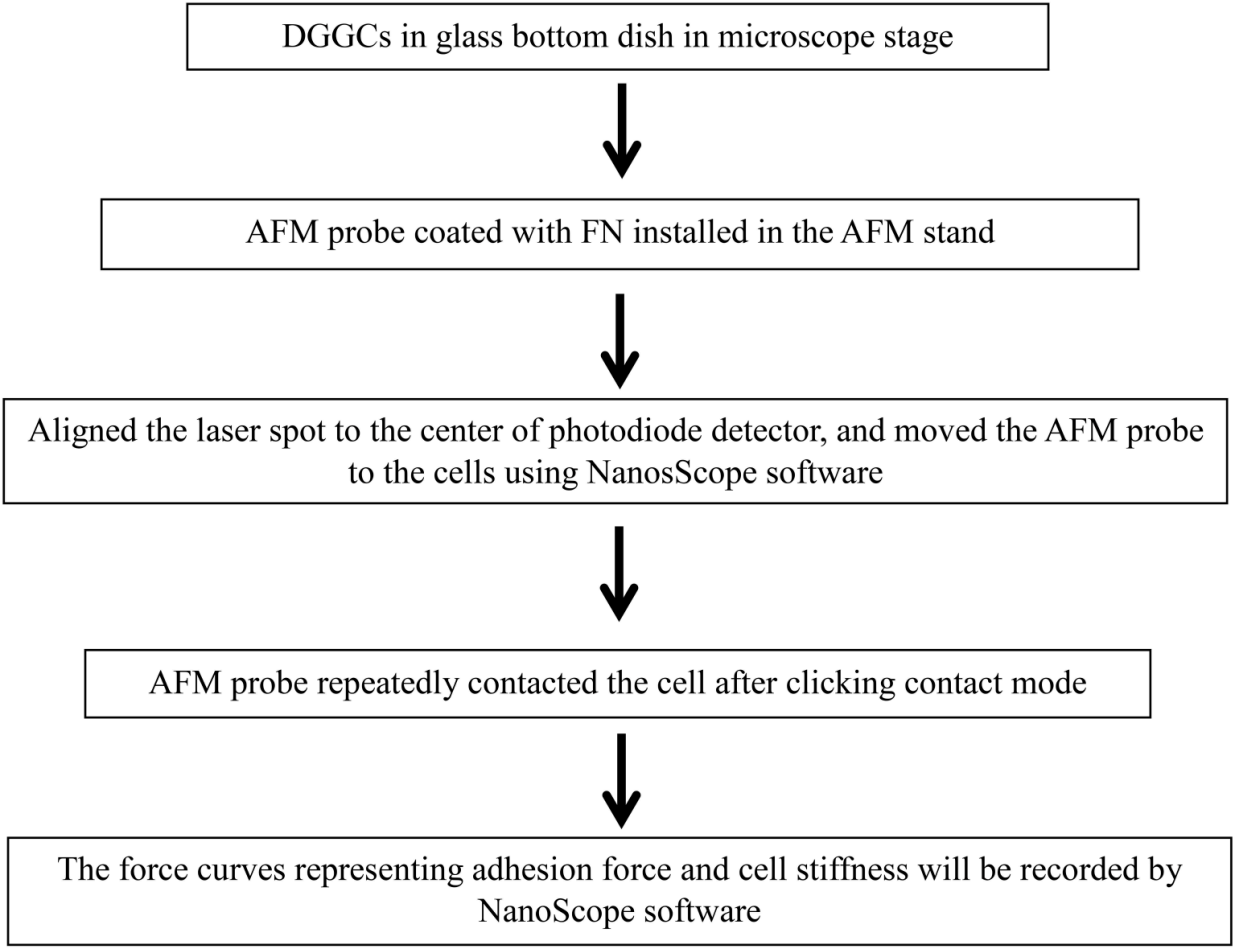

#### Labeling of AFM probes with ECM protein fibronectin (FN)

For adhesion force measurement, the AFM probes are usually coated with the ligands of interest (e.g., FN) to permit the study of ligands and their specific surface receptor interaction (Wu et al., 1998, 2010a; Sun et al., 2005, 2008; Tangney et al., 2013b). The silicon nitride cantilever tip is first rinsed with acetone and let to air-dry for 1 min. Then the probe was installed on AFM cantilever holder (Figure 1, f). The polyethylene glycol (PEG, Sigma) was used to cross-link proteins onto silicon nitride tip at room temperature. The next step is to incubate the AFM probe tip with 30 μl 10 mg/ml PEG for 5 min, and then carefully remove the PEG, wash with phosphate buffered saline (PBS) 4 times, and incubate again with 20 μl 1 mg/ml FN for 60 s, then wash the tip with PBS 4 times. For biotin-labeled borosilicate bead AFM probe, 20 μl FN is added to the probe for 5 min, then wash the probes 5 times with PBS (Wu et al., 1998).

#### AFM maneuver with nanoscope III software

**Figure 3:**
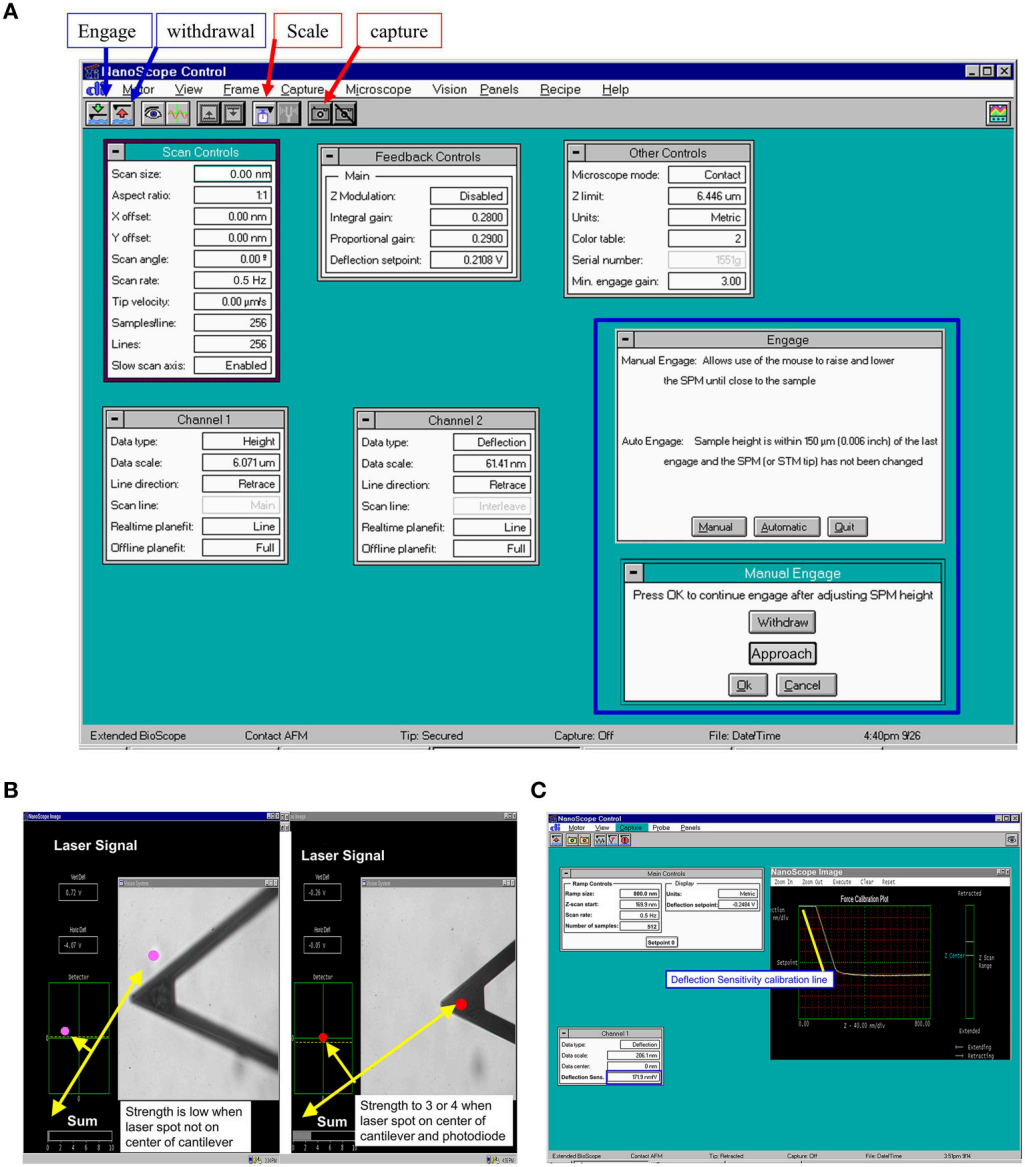
**Representative program screen shot illustrations**. **(A)** AFM scan control in main NanoScope control screen with manual engage window (inside blue frame). **(B)** Laser signal changes during alignment of laser beam on AFM cantilever. Laser signal will be stronger when laser spot focuses on the center of cantilever and center of photodiode. **(C)** AFM probe main control and raw force curve during recording. The yellow line is drawn for measuring the AFM tip deflection sensitivity and sensitivity will be shown in the deflection sensitivity cell with blue frame in Channel 1 setting.

1. Put a 60 mm glass bottom culture dish with one drop of the freshly isolated DGGCs in 2 ml physiological bath solution for at least 30 min on the inverted microscope stage (equipped with 32 × lens). All AFM experiments were performed at 22–24°C.
2. Start Nanoscope software (version 5.12. Notes 5). Select the “microscope icon” in the “NanoScope control” window (Figure 3A). Turn on AFM system Conditioner, optic illuminator and the video camera box controller (Figure 1A).
3. Install the AFM probe (Figure 1B, h) to the clear plastic AFM probe holder (Figure 1B, f).
4. Prepare the AFM probe cantilever with 1 drop of acetone, dry it, wash it with PBS 3 times, and coat the AFM probe with extracellular matrix protein fibronectin (FN) (see Labeling of AFM Probes with ECM Protein Fibronectin (FN)).
5. After labeling with FN, mount the plastic probe holder onto the AFM Scanner (AFM head in Figure 1A) with the O-ring seal on (Figure 1B, e) to prevent a short circuit by the PSS solution (Notes 6).
6. Mount and secure the AFM Scanner to the position for AFM in microscope stage through one adjustable screw (Figure 1B, i).
7. Align the laser beam, so that any deflection of AFM cantilever will be detected by photodiode detector through movement of the laser beam. To *bring the laser onto the cantilever* (Figure 1B, h; Figure 3B) and bring the bright red laser light indicator shown in the (photodiode detector) window (Figure 1B, j; Figure 2A), gently turning the top two screws (Figure 1B, a, b) on the AFM Scanner. In addition, a white bar will show the strength of laser light signaling in the “Sum” in “NanoScope image” window of the program (Figure 3B). Carefully adjust the two screws (Figure 1B, c, d) on the side of AFM Scanner to *bring laser onto center of cantilever and center of the photodiode diagram* that displayed with maximum signal bar (“Sum” normally >3, Figure 3B).
8. Select “Microscope” icon in “NanoScope control,” click “reset.” The 4 red lights on electronic box (Figure 1A) will be off now, and the green light will start flickering.
9. Fine-tune the focus to observe the cell, and then move the focus to well above the cell surfaces.
10. Click the green “DOWN” arrow button in “NanoScope control” to lower the AFM Scanner down to the liquid, select “Manual” and press on “Approach” to lower the cantilever down into the buffer, and finally lower it into the focal plane (Figure 3A, insert in blue frame) (Notes 7). Use the *x*- or *y*-axis knob (in the stage of microscopy) to bring the tip of cantilever to the blue crosshair in the center of video monitoring (“vision system”) window in the program.
11. Make sure the laser spot is still in the center of the photodiode diagram with fine adjustment of screw “c” and “d” in Figure 1B.
12. Before collecting the cell force curve data, the AFM probe sensitivity has to be checked using AFM tip to touch and withdraw from the non-cell region (Notes 8). The operating steps are similar to step 13 through 19. Check the sensitivity by drawing a line parallel with the force curve in points 2-3 as described in Figure 2B. The deflection sensitivity should change automatically in the “Channel 1” frame of the “NanoScope Control” Window (Figure 3C, blue frame). Record this number in your notebook for future data analysis.
13. Fine-tune the focus until the cells are clearly seen using the joy stick (Figure 1A, stage controller). Use the *x*- or *y*-axis knob (in the stage of microscopy) to move a cell into the center of the view.
14. Change configurations as Figure 3A for contact force mode on the AFM “NanoScope control” window of the program. The example values are scan size = 0, scan rate = 0.5 Hz, and deflection setpoint = −0.2 to vertical deflection.
15. Repeat step 10, and keep clicking on “Approach” button until the AFM probe is lowered to the cell surface, but the cantilever still appears unfocused.
16. Select “OK” in “Manual Engage” window, the step motor will automatically bring the tip down to the cell. AFM will automatically start scanning the cells in Contact Mode when the AFM tip senses the deflection equal to deflection setpoint.
17. To generate a topographic image of the cell surface, set the instrument to apply a constant force on the cell. In each horizontal line scan, record both height data (z axis) and the position of the probe (deflection data) in the “NanoScope image” window. At this point, we skip this step and proceed to next step since we only discuss constant force mode in this article.
18. Press on the “Scale” button in “NanoScope control,” the force curve mode operation will be started. The real-time force curves will be shown in the window of “NanoScope image” (Figure 3C).
19. Press on “Setpoint 0” to reset the force curve into the center of y-scale, adjust the force curve position on the x-scale by moving the “Z-Scan Start” in “Main Controls” window back and forth. Modify the Scan rate and ramp size (e.g., 800 nm) as needed (Figures 3A,C).
20. To continuously record force curves (Video 1), press “Capture” in the menu bar on “NanoScope control” window and click “continue.” To stop recording, click “Capture” menu again, and then click “abort.”
21. Press the “Eye” icon on the window of “NanoScope control” to return AFM into contact mode imaging.
22. Press the withdraw button (red “UP” arrow button, Figures 3A,C) to withdraw the AFM head from the cell.
23. To lift the AFM probe out of the solution, keep pressing the withdraw button. To raise the AFM Scanner from the stand, tighten the screw (Figure 1B, **i**) on stand to unlock AFM Scanner, carefully detach the O-ring and the AFM probe holder from AFM Scanner. Release the locking screw (Figure 1B, **i**) and put the AFM Scanner in the lock position on the stand.
24. Turn off the equipment as reversed on Step 2 including exit the Nanoscope software (Notes 9) and turn off the computer.
25. Use forceps to remove the AFM probe from the holder on the black holder stand, wash the probe holder and O-ring with Contrad 70 and double-distilled water.
26. The holes in the AFM probe holder are dried with Kimwipes, a nonabrasive and low-lint paper. Leave the AFM probe holder dry on the black holder stand for next time use.

### Data analysis

To measure cell membrane stiffness (i.e., elasticity. Figure 2B), the approach force curves will be used (black line). Fit the approach force curves with the Hertz Model between points 2 and 3 using MATLAB software (Mathwork, Inc.) or use NForceR software (copyright, 2004) to calculate the cortical stiffness based on tip displacement and membrane indentation as described by Wu and colleagues (Wu et al., 2012b). Use the retraction or withdrawal curve (red-line) to analyze the specific adhesion forces related to bonding between AFM tip and cell surface as described previously (Wu et al., 2010a). During retraction, if a specific adhesion event occurs, it will be detected as small sharp shift (bond rupture, point 5) in the deflection curve obtained during probe retraction from the cell surface. No adhesions will be apparent as a smooth retraction curve similar in appearance to the approach curve. These deflection shifts in withdrawal curve, referred to as snap-offs, will be recorded. The “snap-offs” or rupture force represents the force expected to cause adhesive binding breakdown between the ligands coated at AFM probe and receptor in a cell surface (e.g., FN and α5β1-integrin receptor), and is termed as adhesion force (Shroff et al., 1995; Sun et al., 2005). All snap-offs or adhesion force between FN and its integrin receptor on DGGCs will be collected and charted as a function of the rupture events. Hooke's Law will be used to determine the adhesion force (i.e., rupture forces or unbinding force):

F (adhesion force)=k∗d

Where *k* is the spring constant of the AFM probe cantilever and *d* is the height of the snap-offs in the withdrawal curve (in Figure 2B, point “5”).

#### Data analysis

Group averaged data are represented as means ± S.E.M. Statistical evaluations are achieved with repeated-measures analysis of variance (ANOVA) followed by *post-hoc* tests, or with independent two-tail *t*-tests, as appropriate. The results are considered to be statistically significant if *p* values are < 0.05.

## Anticipated results and discussion

In this protocol, we successfully utilized and optimized the AFM technique for the measurements of neuronal membrane elasticity, and related dynamics that are found to be drastically altered in epilepsy. AFM probe coated with FN is employed to the DGGCs to measure the adhesion force and the cell membrane elasticity between FN and integrin receptor in the DGGC surface. Force curves are collected through continual approach and withdrawal cycles of the AFM probe at certain scan rate (e.g., 0.5 Hz) and z-axis movement as described by Wu and colleagues (Wu et al., 2012b). During the FN-coated AFM probe travels to reach the DGGC cell membrane (Figure 2B, black “approach” line, point 1–2), the curve remains flat. After contacting the cell surface, the cantilever will be bent because of the cell membrane elasticity and the position of laser beam will be changed on detector (point 2–3). Point 2 represents a “reflection point or contact point.” Data in the region of points 2–3 are used to fit using Hertz model to calculate the cell cortical stiffness/elasticity. The stiffer the cell, the less the indentation and the steeper the upslope of the force curve (such as 3′ represents glass surface). As the probe retraction starts (red “retraction” line), the resistance force will be decreased (point 3–4). The snap-off that represents a bond rupture, termed adhesion force, between AFM tip and the DGGCs is shown in the retraction line (red-line point 5). As seen in Figure 2B, the example trace shows 2 adhesion events (bond rupture) that occurred when the FN coated-probe retracted. When all adhesions between the FN-coated probe and DGGC have been broken, the retraction curve again overlies the initial approach curve level (point 6). The data shown here was recorded while the AFM tip located 25% away from the boundary of the neurons body. Some additional technical notes are listed in Box 2.

Box 2 **Additional technical notes on AFM protocol.**

**Protocol Notes:**

1. When working with isoflurane, always work under a fume hood.
2. For a great yield of healthy cells: (a) remove the brain tissue from the anesthetized mouse as quick as possible and then put in ice-cold solution; (b) the incubation time 23–25 min at a temperature of 24°C should be strictly followed; (c) young adult mice (2–3 months) are better than older animals (>6 months).
3. Carefully isolate hippocampal subfields under dissecting microscope and transfer brain slices to Brain Slice Keeper.
4. Don't over triturate because it will damage the freshly isolated cells.
5. Always turn on computer first in AFM system.
6. Avoid overfilling the bath solution, there should be no liquid past the AFM probe O-fluid cantilever holder in order to avoid burning the AFM head by short circuit. If the poles touch the liquid, clean and remove the liquid immediately.
7. Do not bring the AFM tip all the way down to the cell during the manual approach; you may damage the probe and/or the cell.
8. Test probe cantilever sensitivity before collecting data by moving among different cells and validating that the AFM probe was not damaged or contaminated during the preparation or approaching processes.
9. Backup your data and put into another secure place for data protection. PC with RAID-enabled system is recommended.

**Figure 4:**
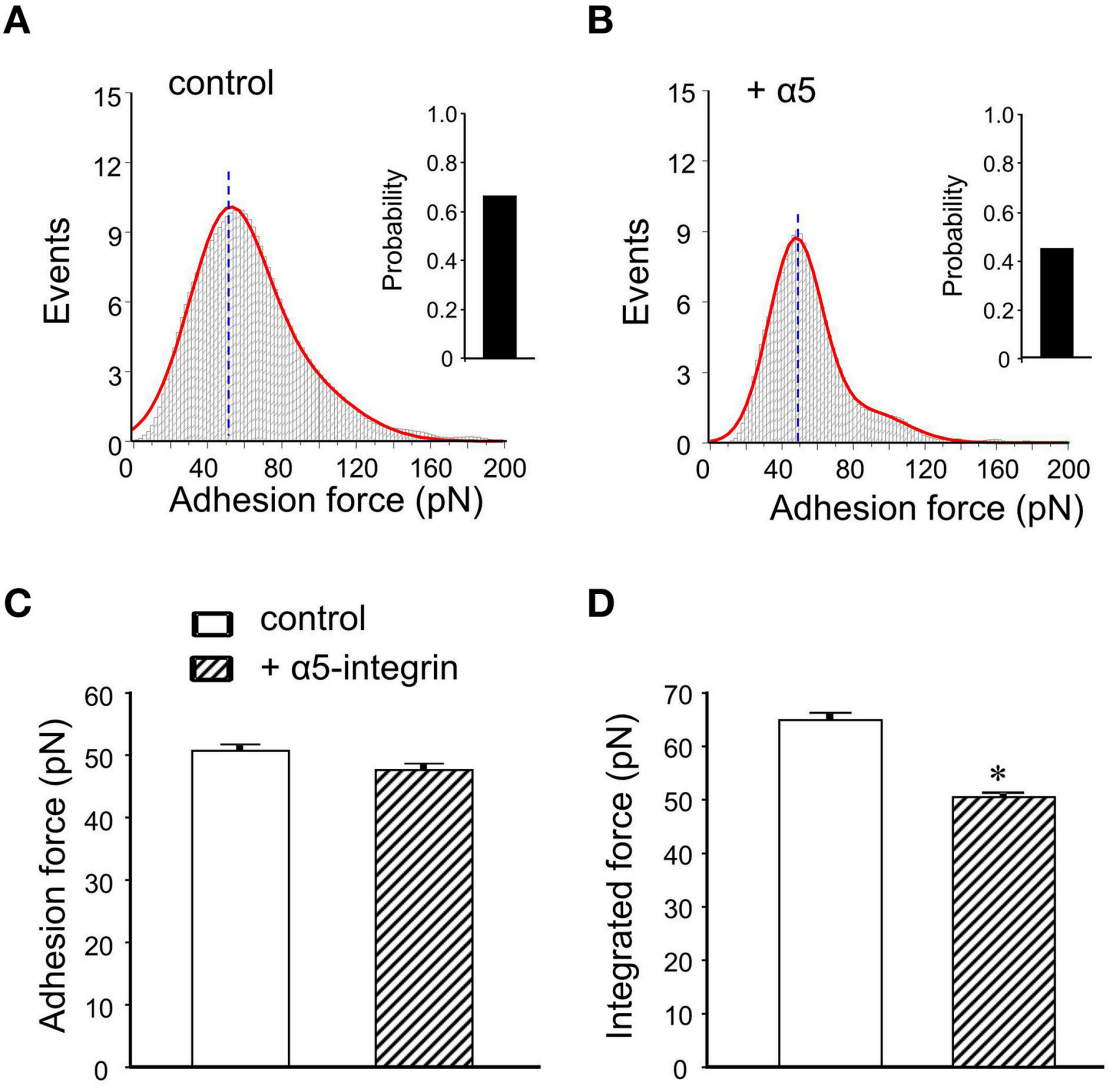
**Summary of adhesion force results with the FN coated AFM probe in mice DGGCs. (A)** Analysis of adhesion force-adhesion event plots during FN-coated probe retraction. The observed adhesion force and corresponding number of events in the experiments (50 curves/cell for a total of 500 curves) were plotted as histograms. Red line represents the results that fitted with multiple Gaussian distributions. Insets: integrin-FN binding probability (solid bar). **(B)** Force-adhesion event plots and integrin-FN binding probability (solid bar) in the presence of function-blocking antibody against α5-integrin (60 nM). **(C)** Summary of the adhesion force that represents the first peak force. **(D)** Summary of the integrated force that represents the total area under the force-events distribution curves. Adhesion force was not changed in the presence of α5-integrin monoclonal antibodies. Integrated force, which provides a metric reflecting the average overall adhesiveness, was decreased by α5-integrin monoclonal antibodies. ^*^P < 0.05 vs. DGGCs in control (FN-coated probe alone). *n* = 10 for each group.

To calculate adhesion force between FN and its integrin receptor, the distribution of adhesion force and the observed “snap-offs” events in the retraction curve were analyzed with Gaussian distributions. The data revealed a good covenant between the original data (gray histogram) and the fitting line (Figure 4A, red line). The initial peak of the FN-integrin single bond unbinding force (i.e., adhesion force; Sun et al., 2005; Wu et al., 2010a) was about 50.7 ± 1.4 pN (*n* = 10 cells from 3 to 4 mice). Previous works in our laboratories and by others have reported that the adhesion force between FN and α5β1-integrin is between 35 and 80 pN (Li et al., 2003; Sun et al., 2005; Trache et al., 2005). The bar graph in the right margin of the Figure 4A showed the probability of adhesion between FN-integrin receptor. The probability of adhesion events, expressed as the percentage of the force curves with snap-offs divided by total curves collected, was 66% in control mice group. The α5β1-integrin has been documented to bind FN (Wu et al., 1998, 2010a). Since α5-integrin subunit was associated only with the β1-integrin subunit (Hynes, 1992), the anti-α5-integrin monoclonal antibody (60 nM) was used to block FN binding to α5β1-integrin subunits. The initial peak of the adhesion force had no significant change after application of anti-α5-integrin in the bath solution (47.7 ± 0.4 pN, *n* = 10; Figures 4B,C). However, the adhesive probability was decreased by 31% after application of anti-α5-integrin (Figure 4B).

The integrated force value was verified as averaged force from all adhesion events. Pretreatment with antibody exhibited less spread under the adhesion force density distribution curve than with FN alone (i.e., reduced the area). It indicated less total adhesion events (Figures 4A,B). The integrated adhesion force between FN and the DGGCs was 63.8 ± 1.2 pN and was reduced by 20% in the presence of α5-integrin monoclonal antibody (Figure 4D). The data from this study indicated that the adhesion probability to integrins significantly declined, but not the adhesion force in the presence of α5β1-integrin monoclonal antibody. These results are similar to our previous observation in cardiomyocytes (Wu et al., 2010a). The results above also indicated that α5-integrin monoclonal antibody, as a competitive inhibitor reduced the availability of integrin to the FN, presumably acted by inhibiting FN from interacting with integrin on the cell surface. Therefore, the adhesion probability and integrated force between FN-integrin were decreased. These data supported the α5β1-integrin specificity of the binding to FN.

**Figure 5:**
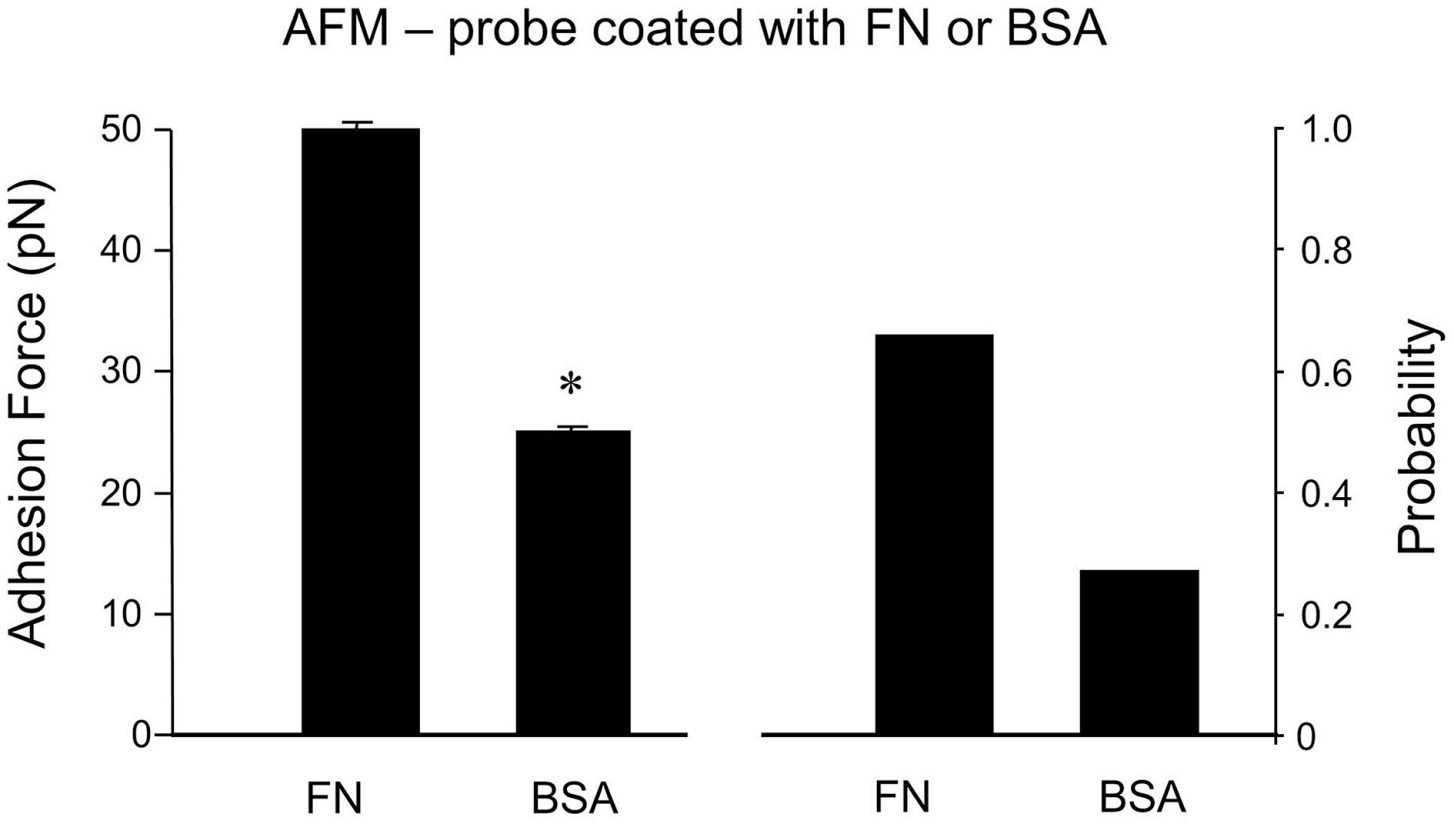
**Specificity of adhesion force in DGGCs by FN**. The peaks of adhesion force and binding probability using bovine serum albumin (BSA)-coated AFM probes as non-integrin ligands were significantly smaller than that using FN-coated AFM probes. ^*^P < 0.05 vs. DGGCs in FN. *n* = 10 for each group.

As a non-specific protein control, bovine serum albumin (BSA)-coated FM probes were examined. BSA showed a significantly reduced adhesion probability and adhesion force with cell membrane compared to FN (−59% and −55%, respectively; *n* = 10; Figure 5). This confirms that the adhesion force between FN and integrin receptor in the cell membrane is specific binding.

**Figure 6:**
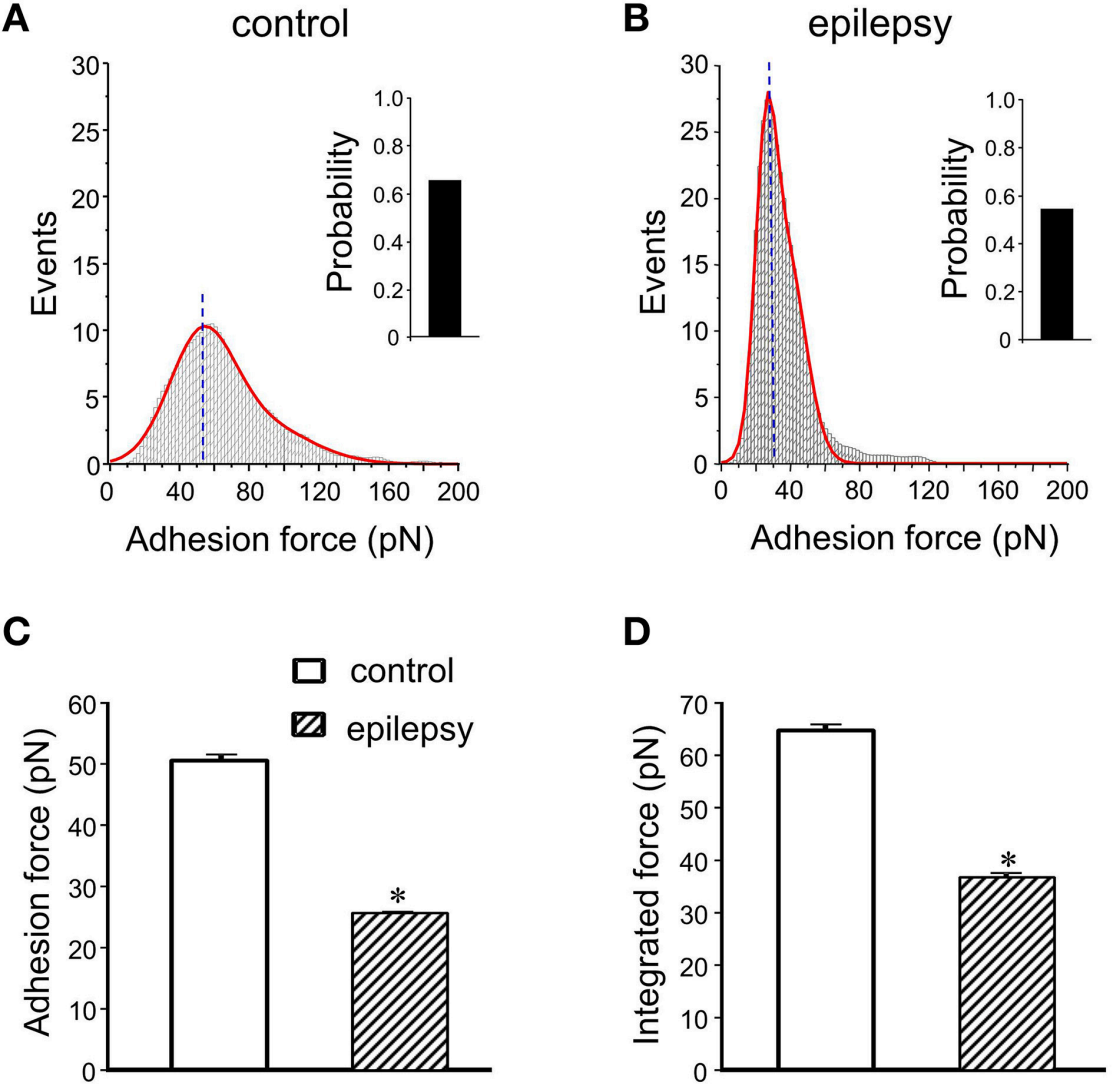
**Summary data of adhesion force and integrated force in DGGCs from epilepsy**. **(A,B)** Analyses of adhesion force-adhesion event plots during FN-coated probe retraction in DGGCs from control and epilepsy mice (stage 5 kindling). **(C,D)** Summary of normalized results of adhesion characteristics of DGGCs. Adhesion force (C. 25.6 ± 0.2 pN) and integrated force **(D)** were significantly decreased in epilepsy mice. ^*^P < 0.05 vs. DGGCs in control mice. *n* = 10 for each group.

**Figure 7:**
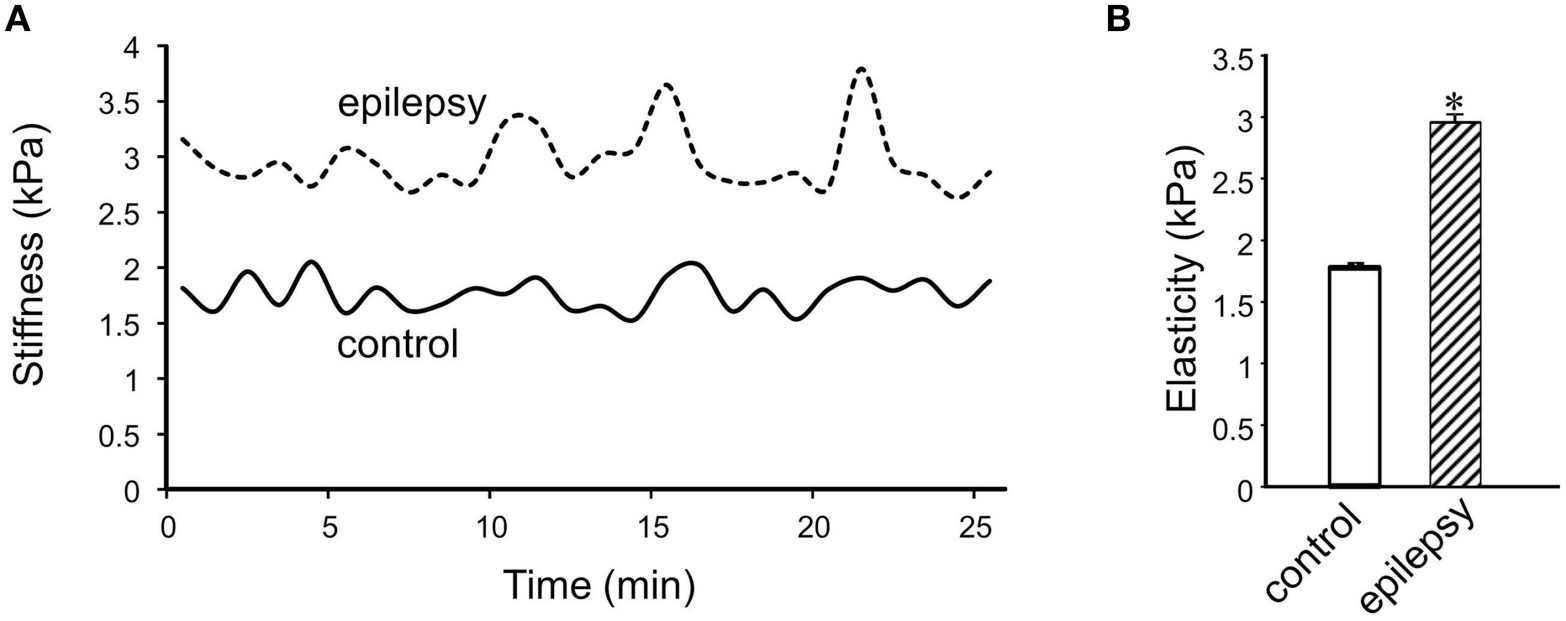
**Measurement of cell plasticity/elasticity with AFM in DGGCs**. **(A)** The time course of elastic modulus in DGGCs from control and epilepsy mice with FN-coated AFM probe. The elastic modulus of DGGCs from stage 5 epilepsy was increased comparing to control mice and no time dependence. **(B)** Bar graph summary of stiffness changes in epilepsy mice (*n* = 10). The cell elasticity/plasticity was increased in stage 5 epilepsy mice. ^*^P < 0.05 vs. DGGCs in control mice. *n* = 10 for each group.

In DGGCs from stage 5 epileptic mice, the adhesion force and adhesive probability between FN and cell surface were significantly lower (−49% and −23%, respectively) when compared to control mice (*n* = 10; Figure 6). The integrated force was decreased by 42% in DGGCs from epileptic mice. To quantitatively calculate the cell stiffness or membrane elasticity, the portion between points 2 (deflection point or contact point) and 3 in the approach curve (Figure 2B) was analyzed. Figure 7A showed the continuous changes in the stiffness/elasticity values during time course for cells from control and epileptic mice. The stiffness in epileptic mice was high in all given time and no-time dependence. The average value of cell stiffness after FN coated probe approached the cell membrane at 1.77 ± 0.03 kPa (Figure 7B). In epileptic mice, the cell stiffness showed a significant increase (2.96 ± 0.07 kPa). It has been suggested that increase in stiffness is associated with changes in integrin expression, [Ca^2+^]_*i*_levels and activation of cytoskeletal filaments (Paul et al., 2000; Rueckschloss and Isenberg, 2004; Wu et al., 2010a). The changes of cell elasticity might be associated to cell remodeling, dispersion of the DGGC layer and the appearance of neurons in ectopic locations during development of epilepsy.

In epilepsy, increased integrin expression and increased apoptotic cell death and neuronal proliferation in the DGGCs, principal excitatory phenotype neurons, cause hyper-synchronization leading to development of epilepsy (Gall and Lynch, 2004; Kokaia, 2011). During epileptogenesis in both human and rodent, DGGCs undergo extensive remodeling, including reorganization of mossy fibers, dispersion of the DGGC layer, and the appearance of DGGCs in ectopic locations within the dentate gyrus. Integrin and FAC recruitment, integrin-ECM detachment and attachment have been reported to dynamically change their position *at leading and trailing edges* in migrating cells (Becchetti and Arcangeli, 2010; Huttenlocher and Horwitz, 2011). In order for DGGCs to spread or re-localize within the hippocampus they need to modify their anchoring positions to the ECM (binding change) and their cytoskeletal architecture (cell elasticity change). Cleavage of adhesive connections (i.e., unbinding) and changing cell shape for migrating (i.e., elasticity change) are early steps in the formation of new synaptic configurations (Chang et al., 1993). To gain a fundamental understanding of epilepsy related changes in DGGCs at nanoscale resolution, it is necessary to first ask how cells attach, spread, and migrate through dynamic integrin receptor activation. Our results indicate that changes in adhesion force and probability, as well as cell membrane elasticity, may contribute to epileptogenesis.

In conclusion, these results suggest that the AFM is a cutting-edge nanotechnique for studies of dynamic membrane plasticity and its progressive alteration with brain injury or disease. Our AFM data showed that FN-integrin interactions in DGGCs drastically modulate adhesion force and membrane elasticity in epilepsy mice. Thus, the AFM method provides a unique tool for molecular investigations of neuronal membrane dynamics in a variety of neurological diseases and brain injury models.

## Author contributions

Participated in research design: XW, MM, DR. Conducted experiments: XW, DR. Performed data analysis: XW, DR. Wrote or contributed to the writing of the manuscript: XW, MM, DR.

### Conflict of interest statement

The authors declare that the research was conducted in the absence of any commercial or financial relationships that could be construed as a potential conflict of interest.

## Supplementary material

Video 1 **Represents contact force mode recording (available in online version)**. Raw force curves generated using FN-coated AFM probe (also see Figure 2 for details). FN-coated cantilever (1 mg/ml) were controlled to repeatedly approach (black trace) and retract (red trace) from cell while *xy*-axes fixed (right panel). The snap-off that represents bond rupture, termed adhesion force (left panel raw red traces). The example trace shows 3 adhesion events (bond rupture) between FN ligand (gradient brown circle under gray AFM probe in right panel) and 3-integrin receptors (gradient blue Y-shape) in the cell that occurred when the FN coated-probe retracted.
